# First Principles Calculation of the Entropy of Liquid Aluminum

**DOI:** 10.3390/e21020131

**Published:** 2019-01-31

**Authors:** Michael Widom, Michael Gao

**Affiliations:** 1 Department of Physics, Carnegie Mellon University, Pittsburgh, PA 15217, USA; 2 National Energy Technology Laboratory, Albany, OR 97321, USA; 3 Leidos Research Support Team, P.O. Box 10940, Pittsburgh, PA 15236, USA

**Keywords:** Gibbs entropy, Shannon entropy, liquid metal

## Abstract

The information required to specify a liquid structure equals, in suitable units, its thermodynamic entropy. Hence, an expansion of the entropy in terms of multi-particle correlation functions can be interpreted as a hierarchy of information measures. Utilizing first principles molecular dynamics simulations, we simulate the structure of liquid aluminum to obtain its density, pair and triplet correlation functions, allowing us to approximate the experimentally measured entropy and relate the excess entropy to the information content of the correlation functions. We discuss the accuracy and convergence of the method.

## 1. Introduction

**Figure 1:**
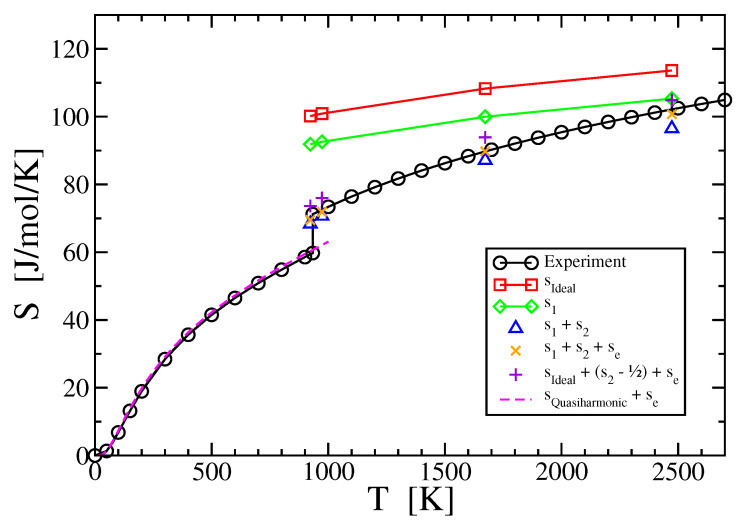
Calculated entropies compared with experimental values [6,7]. $s_{\mathrm{Ideal}}$ is from Equation (12), $s_{1}$ is from Equation (11), $s_{2}$ is the pair-correlation correction from Equation (14), and $S_{e}$ is from Equation (27)). We expect the best liquid state result from $s_{1}+s_{2}+s_{e}$. In the solid state, below melting at T${}_{m}$ = 933 K, $s_{\mathrm{Quasiharmonic}}$ is the vibrational entropy in the quasiharmonic approximation.

Let $p_{i}$ be the probability of occurrence of a state *i*, in thermodynamic equilibrium. The Gibbs’ and Von Neumann’s formulas for the entropy [1,2],

(1) $$S/k_{B}=-\sum_{i}p_{i}\ln p_{i},$$

are mathematically equivalent to the information measure defined by Shannon [3]. Entropy is thus a statistical quantity that can be calculated without reference to the underlying energetics that created the probability distribution, as recognized by Jaynes [4]. Previously we applied this concept to calculate the entropy of liquid aluminum, copper and a liquid aluminum-copper alloy binary alloy [5], using densities and correlation functions obtained from first principles molecular dynamics simulations that are nominally exact within the approximations of electronic density functional theory. In this paper we discuss the convergence and principal error sources for the case of liquid aluminum. As shown in Figure 1, we are able to reproduce the experimentally known entropy [6,7] to an accuracy of about 1 J/K/mol, suggesting that this method could provide useful predictions in cases where the experimental entropy is not known.

In a classical fluid [8], the atomic positions $r_{i}$ and momenta $p_{i}$ (i=1,…,N for *N* atoms in volume *V*) take a continuum of values so that the probability becomes a function, $f_{N}\left(r_{1},p_{1},\ldots ,r_{N},p_{N}\right)$, and the entropy becomes

(2) $$S_{N}/k_{B}=-\frac{1}{N!}\int_{V}\prod_{i}dr_{i}dp_{i}\;f_{N}\ln\left(h^{3N}f_{N}\right)$$

in the canonical ensemble. In this expression, the factor of N! corrects for the redundancy of configurations of identical particles, and the factors of Planck’s constant *h* are derived from the quantum mechanical expression. For systems whose Hamiltonians separate into additive terms for the kinetic and configurational energies, $f_{N}$ factorizes into a product $\prod_{i}f_{i}g_{N}^{\left(N\right)}$ of independent Maxwell-Boltzmann distributions for individual atomic momenta,

(3) $$f_{1}\left(p\right)=\rho {\left(2\pi mk_{B}T\right)}^{-3/2}e^{-{\left|p\right|}^{2}/2mk_{B}T},$$

times the *N*-body positional correlation function $g_{N}^{{(}^{)}}N\left(r_{1},\ldots ,r_{N}\right)$.

Equation (2) can be reexpressed in terms of *n*-body distribution functions [8,9,10,11], $g_{N}^{\left(n\right)}$ with n<N, as

(4) $$S/Nk_{B}=s_{1}+s_{2}+s_{3}+\ldots ,$$

where the *n*-body terms are

(5) $$\begin{array}{cc} s_{1} & =-\frac{1}{\rho }\int_{V}dp\;f_{1}\left(p\right)\ln\left(h^{3}f_{1}\left(p\right)\right) \end{array}$$

(6) $$\begin{array}{cc} s_{2} & =-\frac{1}{2}\rho^{2}\int_{V}dr_{1}r_{2}\;g_{N}^{\left(2\right)}\ln g_{N}^{\left(2\right)}, \end{array}$$

(7) $$\begin{array}{cc} s_{3} & =-\frac{1}{6}\rho^{3}\int_{V}dr_{1}r_{2}r_{3}\;g_{N}^{\left(3\right)}\ln\left(g_{N}^{\left(3\right)}/g_{N}^{\left(2\right)}g_{N}^{\left(2\right)}g_{N}^{\left(2\right)}\right). \end{array}$$

The subscripts *N* indicate that the correlation functions are defined in the canonical ensemble, with a fixed number of atoms *N*, and they obey the constraints

(8) $$\rho^{n}\int_{V}\prod_{i}dr_{i}\;g_{N}^{\left(n\right)}=\frac{N!}{(N-n)!}.$$

Each term $s_{n}$ can be interpreted in terms of measures of information. Briefly, $s_{1}$ is the entropy of a single particle in volume V=1/ρ, and hence in the absence of correlations. $s_{2}$ is the difference between the information content of the pair correlation function $g_{N}^{\left(2\right)}$, and the uncorrelated entropy, which must be added to $s_{1}$. Similarly, $s_{3}$ is the difference between the information contents of the three-body correlation $g_{N}^{\left(3\right)}$ and the two-body correlation $g_{N}^{\left(2\right)}$, which must be added to $s_{1}+s_{2}$. Notice that the information content of the *n*-body is also contained in the (n+1)-body and higher-body correlations because of the identity

(9) $$g_{N}^{\left(n\right)}\left(r_{1},\ldots ,r_{n}\right)=\frac{\rho }{N-n}\int_{V}dr_{n+1}g_{N}^{\left(n+1\right)}\left(r_{1},\ldots ,r_{n},r_{n+1}\right)$$

that expresses $g_{N}^{\left(n\right)}$ as a marginal distribution of $g_{N}^{\left(n+1\right)}$.

Mutual information measures how similar a joint probability distribution is to the product of its marginal distributions [12]. In the case of a liquid structure, we may compare the two-body joint probability density [13,14] $\rho^{\left(2\right)}\left(r_{1},r_{2}\right)=\rho^{2}g_{N}^{\left(2\right)}\left(\right|r_{2}-r_{1}\left|\right)$ with its single-body marginal, $\rho^{\left(2\right)}\left(r\right)$. The mutual information per atom

(10) $$I\left[\rho^{\left(2\right)}\left(r_{1},r_{2}\right)\right]=\frac{1}{N}\int_{V}dr_{1}dr_{2}\;\rho^{\left(2\right)}\left(r_{1},r_{2}\right)\ln\left(\rho^{\left(2\right)}\left(r_{1},r_{2}\right)/\rho \left(r_{1}\right)\rho \left(r_{2}\right)\right)$$

tells us how much information g(r) gives us concerning the positions of atoms at a distance *r* from another atom. Mutual information is nonnegative definite. We recognize the term $s_{2}$ in Equation (6) as the negative of the mutual information content of $g_{N}^{\left(2\right)}$, with the factor of 1/2 correcting for double-counting of pairs of atoms.

## 2. General Theory

### 2.1. One-Body Term

The one-body term $s_{1}$ in Equation (5) can be evaluated explicitly, yielding

(11) $$s_{1}=\frac{3}{2}-\ln\left(\rho \Lambda^{3}\right),$$

where $\Lambda =\sqrt{h^{2}/2\pi mk_{B}T}$ is the quantum De Broglie wavelength. Both terms in Equation (11) have simple information theoretic interpretations [15]. While an infinite amount of information is required to specify the exact position of even a single particle, in practice, due to quantum mechanical uncertainty we should only specify position with a resolution of Λ. Consider a volume V=1/ρ. In the absence of other information, the probability that a single particle is localized within a given volume $\Lambda^{3}$ is $p=\Lambda^{3}/V$. Summing −plnp over the ($V/\Lambda^{3}$)-many such volumes yields $-\ln\left(\Lambda^{3}/V\right)=-\ln\left(\rho \Lambda^{3}\right)$. Similarly, the 3/2 in Equation (11) is simply the entropy of the Gaussian momentum distribution, Equation (3).

Notice that $s_{1}$ resembles the absolute entropy of the ideal gas,

(12) $$S_{\mathrm{Ideal}}=\frac{5}{2}-\ln\left(\rho \Lambda^{3}\right).$$

The difference lies in the constant term 3/2 in $s_{1}$ *vs.* 5/2 in $S_{\mathrm{Ideal}}$. We shall discover that the difference 5/2−3/2=1 is accounted for in the many-body terms $s_{2},s_{3},\ldots$. Indeed, this is clear if we place *N* particles in the volume V=N/ρ. The derivation of Equation (11) generalizes to $s/Nk_{B}=\frac{3}{2}-\ln\left(\Lambda^{3}/V\right)$, but this must be corrected [15] by the irrelevant information, lnN!, that identifies the individual particles in each separate volume $\Lambda^{3}$. The leading term of the Stirling approximation lnN!≈NlnN−N converts $\ln\left(\Lambda^{3}/V\right)$ into $\ln\left(\rho \Lambda^{3}\right)$, while the second term adds 1 to 3/2 yielding 5/2.

Either $s_{1}$ or $s_{\mathrm{Ideal}}$ can be taken as a starting point for an expansion of the entropy in multi-particle correlations. Prior workers [11,16,17,18,19] tend to favor $s_{\mathrm{Ideal}}$, while we shall find it more natural to begin with $s_{1}$.

### 2.2. Two- and Three-Body Terms

Translational symmetry allows us to replace the double integral over positions $r_{1}$ and $r_{2}$ in Equation (6) for $s_{2}$ with the volume *V* times a single integral over the relative separation $r=r_{2}-r_{1}$. A similar transformation applies to the integral for $s_{3}$. However, the canonical ensemble constraint Equation (8) leads to long-range (large *r*) contributions to the remaining integrations. Nettleton and Green [16] and Raveche [17,18] recast the distribution function expansion in the *grand*-canonical ensemble and obtained expressions that are better convergent. We follow Baranyai and Evans [11] and apply the identity

(13) $$\rho^{2}\int_{V}dr_{1}dr_{2}\;g_{N}^{\left(2\right)}\left(r_{1},r_{2}\right)=N\left(N-1\right)$$

to rewrite the two-body term as

(14) $$\begin{array}{cc} s_{2} & =S_{\mathrm{Fluct}}^{\left(2\right)}+S_{\mathrm{Info}}^{\left(2\right)} \end{array}$$

(15) $$\begin{array}{cc} S_{\mathrm{Fluct}}^{\left(2\right)} & =\frac{1}{2}+\frac{1}{2}\rho \int dr\;\left[g^{\left(2\right)}\left(r\right)-1\right] \end{array}$$

(16) $$\begin{array}{cc} S_{\mathrm{Info}}^{\left(2\right)} & =-\frac{1}{2}\rho \int dr\;g^{\left(2\right)}\left(r\right)\ln g^{\left(2)(r\right)}. \end{array}$$

The combined integrand $\left\{\left[g^{\left(2\right)}\left(r\right)-1\right]-g^{\left(2\right)}\left(r\right)\ln g^{\left(2\right)}\left(r\right)\right\}$ of $s_{2}$ falls off rapidly, so that the sum of the two integrals converges rapidly as the range of integration extends to large *r*. Furthermore, the combined integral is ensemble invariant, which allowed us to substitute the grand canonical ensemble radial distribution function g(r) in place of the canonical $g_{N}^{\left(2\right)}$. The same trick applies to the three-body term,

(17) $$\begin{array}{cc} s_{3} & =S_{\mathrm{Fluct}}^{\left(3\right)}+S_{\mathrm{Info}}^{\left(3\right)} \end{array}$$

(18) $$\begin{array}{cc} S_{\mathrm{Fluct}}^{\left(3\right)} & =\frac{1}{6}+\frac{1}{6}\rho^{2}\int dr^{2}\left(g^{\left(3\right)}-3g^{\left(2\right)}g^{\left(2\right)}+3g^{\left(2\right)}-1\right) \end{array}$$

(19) $$\begin{array}{cc} S_{\mathrm{Info}}^{\left(3\right)} & =-\frac{1}{6}\rho^{2}\int dr^{2}g^{\left(3\right)}\ln\left(g^{\left(3\right)}/g^{\left(2\right)}g^{\left(2\right)}g^{\left(2\right)}\right). \end{array}$$

The contribution of 1/2 in $s_{2}$ as given by Equation (14), together with an added 1/6+1/12+⋯=1/2 from the three-body Equation (17) and higher terms, reconciles the one-body entropy with the ideal gas. For consistency with previous workers [11,16,17,18,19] who omit the 1/2 from $S_{\mathrm{Fluct}}^{\left(2\right)}$ and the 1/6 from $S_{\mathrm{Fluct}}^{\left(3\right)}$, and to make connection with the ideal gas, we can add the entire series 1/2+1/6+1/12+⋯=1 to $s_{1}$ and write

(20) $$S/Nk_{B}=S_{\mathrm{Ideal}}+\left(s_{2}-1/2\right)+\left(s_{3}-1/6\right)+\cdots$$

which is equivalent to Equation (4).

In the grand-canonical ensemble, the $S_{\mathrm{Fluct}}^{\left(2\right)}$ term in Equation (14) arise from fluctuations in the number of atoms, *N*, and can be evaluated in terms of the isothermal compressibility $\chi_{T}$ as

(21) $$S_{\mathrm{Fluct}}^{\left(2\right)}/k_{B}=\frac{1}{2}\gamma ,$$

where

(22) $$\gamma =\rho k_{B}T\chi_{T}$$

is the dimensionless compressibility. Note that $\chi_{T}$, and hence also ${§}_{\mathrm{Fluct}}^{\left(2\right)}$, are positive definite. The remaining term is the entropy reduction due to the two-body correlation. As noted above, the mutual information content of the radial distribution function $g^{\left(2\right)}\left(r\right)$ reduces the entropy by

(23) $$S_{\mathrm{Info}}^{\left(2\right)}/k_{B}\equiv -\frac{1}{2}\rho \int dr\;g^{\left(2\right)}\left(r\right)\ln g^{\left(2\right)}\left(r\right).$$

The complete two-body term is now $s_{2}=S_{\mathrm{Fluct}}^{\left(2\right)}+S_{\mathrm{Info}}^{\left(2\right)}$.

The three-body fluctuation term (see Equation (16)) also relates to isothermal compressibility [18], with

(24) $$S_{\mathrm{Fluct}}^{\left(3\right)}=\frac{1}{2}\gamma -\frac{1}{3}\gamma^{2}+\frac{1}{6}\rho \gamma \frac{\partial \gamma }{\partial \rho }{|}_{\beta }.$$

The final term in Equation (17) reduces to a difference of three- and two-body entropies, and its sign is not determined. Essentially, the $g^{\left(3\right)}\ln\left(g^{\left(3\right)}/g^{\left(2\right)}g^{\left(2\right)}g^{\left(2\right)}\right)$ term adds back the two-body mutual information $I[g^{\left(2\right)}]$ and then subtracts the information contained in the three-body correlation $g^{\left(3\right)}$. Note that $g^{\left(3\right)}$ necessarily contains all the information in $g^{\left(2\right)}$ because of the identity Equation (9).

The pattern illustrated in Equations (21) and (24) holds for the analogous higher-body correlations as well, because integrals of the correlation function $g^{\left(n\right)}$ can be written in terms of integrals and density derivatives of $g^{\left(n-1\right)}$. One limit of special interest is the incompressible limit, where the initial terms of Equations (14) and (17) vanish and only the information-derived glng terms survive. This limit should apply to dense fluids at low temperatures. Another limit occurs at high temperature, where the density drops and the correlation functions approach 1. In this limit all integrals involving $g^{\left(n\right)}$ vanish so that $S_{\mathrm{Info}}^{\left(n\right)}=0$ and all the $S_{\mathrm{Fluct}}^{\left(n\right)}$ terms sum to 1/2+1/6+1/12+⋯=1.

Truncation of the series of terms $S_{\mathrm{Info}}^{\left(n\right)}$ is accurate if higher many-body correlation functions can be approximated by products of fewer-body correlations. That is, if the higher correlation functions contain no new information. For example, the Kirkwood superposition approximation

(25) $$\delta g_{N}^{\left(3\right)}\left(r,s,t\right)\equiv g^{\left(3\right)}\left(r,s,t\right)/g_{N}^{\left(2\right)}\left(r\right)g_{N}^{\left(2\right)}\left(s\right)g_{N}^{\left(2\right)}\left(t\right)\approx 1$$

causes $S_{\mathrm{Info}}^{\left(3\right)}$ to vanish.

## 3. Results

To provide the liquid state correlation functions needed for our study we perform *ab-initio* molecular dynamics (AIMD) simulations based on energies and forces calculated from first principles electronic density functional theory (DFT). We apply the plane-wave code VASP [20] in the generalized gradient approximation [21]. Simulations are performed at fixed volume for each temperature. In order to determine the proper volumes (i.e., liquid densities ρ) we performed simulations at several volumes to identify the volume at which the pressure (including the kinetic term) vanished. Most runs were performed using Normal precision FFT grids, however the smallest system (*N* = 100 atoms) was found to require accurate precision.

**Figure 2:**
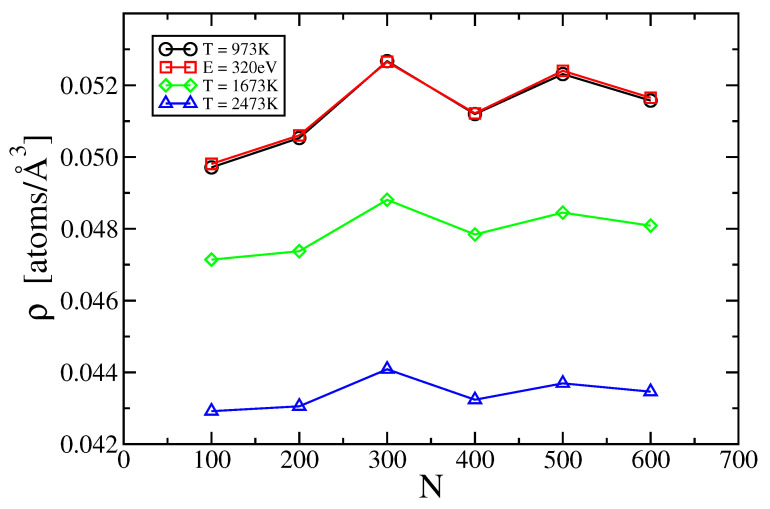
Calculated aluminum density *vs.* number of atoms *N* at various temperatures. All results hold for the default energy cutoff of 240 eV except for red squares that hold for 320 eV.

Figure 2 shows the result of convergence studies in both volume and plane-wave cutoff energy. Briefly, we found minimal dependence on the plane wave energy cutoff, but strong and non-monotone dependence on the number of atoms. We accept N=500 atoms as a suitable target for convergence of the volume and we use the same condition for collecting our correlation functions. Our calculated density at 973 K falls below the experimentally assessed value by about 1%, similar to the discrepancy for solid Al in the limit of low temperature. From the volume-dependence of pressure we obtain estimates of the dimensionless compressibility γ ranging from 0.008 at T = 973 K up to 0.015 at T = 2473 K.

Pair correlation functions $g^{\left(2\right)}\left(r\right)$ are collected as histograms in Δ=0.01 Å bins, normalized to $4\pi r^{2}\Delta N^{2}/V$ and subsequently smeared with a Gaussian of width σ=0.025 Å. Triplet correlation functions $g^{\left(3\right)}\left(r,s,t\right)$ utilize bin widths of Δ=0.10 Å, normalized to $8\pi^{2}rst\Delta^{3}N^{3}/V^{2}$, and are not smeared. Our run durations for data collection were 10 ps. All structures were thoroughly equilibrated prior to data collection.

**Figure 3:**
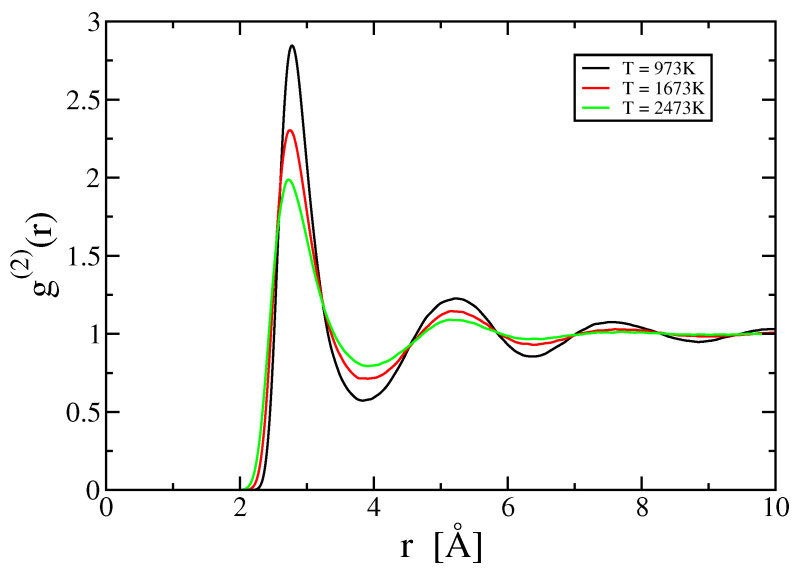
Pair correlation function $g^{\left(2\right)}\left(r\right)$ at various temperatures.

**Figure 4:**
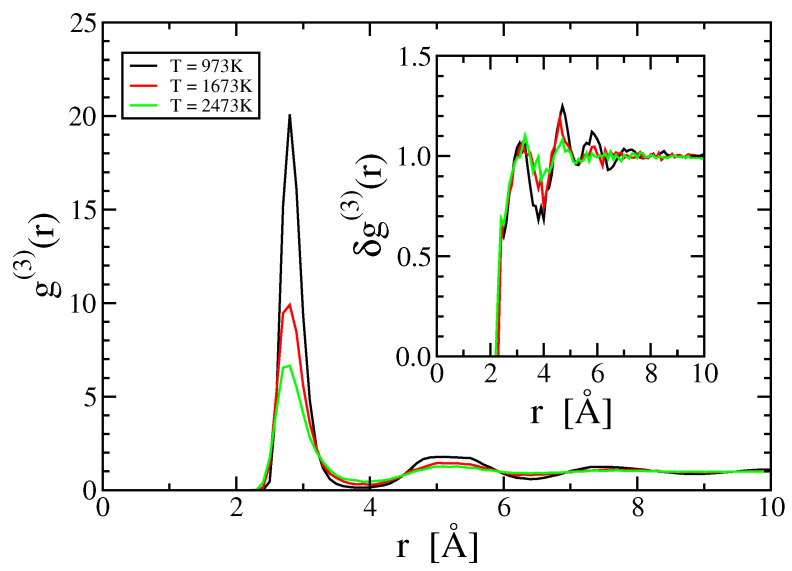
Triplet correlation function $g^{\left(3\right)}\left(r,r,r\right)$ at various temperatures. Inset: Kirkwood ratio Equation (25).

Figure 3 illustrates the pair correlation function $g^{\left(2\right)}\left(r\right)$ at various temperatures. Note the oscillations that extend to large *r*; presumably these oscillations are responsible in part for the oscillations in ρ as a function of *N*. Note also the decreasing amplitude of oscillation with increasing temperature. Figure 4 illustrates the three-body correlation function for the special case of equilateral triangles with r=s=t. The inset displays the ratio $\delta g^{\left(3\right)}\left(r,r,r\right)$ (see Equation (25)). Notice that $\delta g^{\left(3\right)}$ is nearly a step function, with small decaying oscillations that diminish with increasing temperature.

### 3.1. One-Body Term

The one-body term explicitly depends on density, and also depends implicitly on temperature through the De Broglie wavelength Λ. Taking our calculated densities, and evaluating Λ, $s_{1}$, and $s_{\mathrm{Ideal}}$, we note that $s_{1}$ and $s_{\mathrm{Ideal}}$ are greater than, but rather close to, the experimental liquid entropies [6,7], as shown in Figure 1. The differences drop as the temperature grows, as expected because nonideality of the liquid metal becomes less important at high temperature.

### 3.2. Two-Body Term

**Figure 5:**
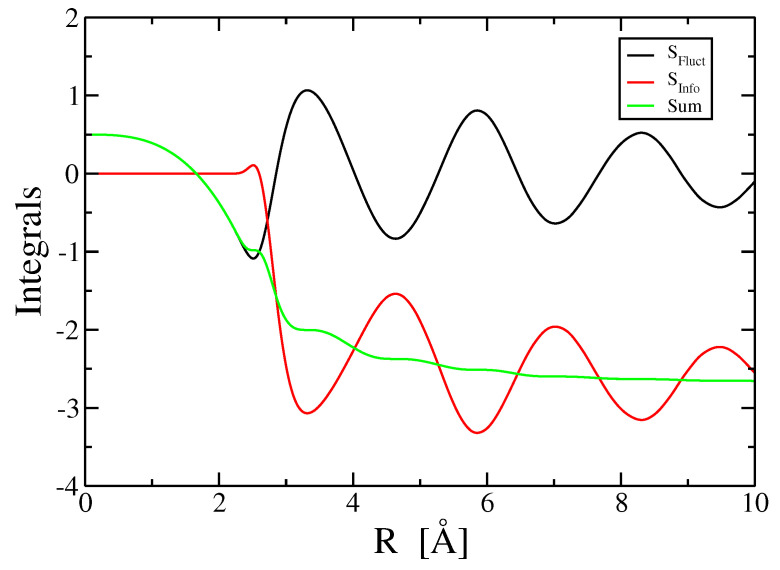
Two-body terms $S_{\mathrm{Fluct}}$ and $S_{\mathrm{Info}}$ and their sum from simulated pair correlation function $g^{\left(2\right)}$ at T = 973 K.

In Figure 5 we plot the terms $S_{\mathrm{Fluct}}^{\left(2\right)}$ and $S_{\mathrm{Info}}^{\left(2\right)}$ as defined by Equations (21) and (23), respectively, where we integrate from zero separation up to a cutoff of *R*. Owing to the $R^{2}$ increase of the volume differential dr, oscillations of $g^{\left(2\right)}$ are magnified at large *R*. The fluctuation term appears to converge towards a value close to 0, consistent with the low compressibility of the liquid metal, while the information term converges towards a negative value. Note that the oscillations are nearly opposites, so that their sum converges rapidly towards a negative value of $s_{2}$.

Adding the entropy reduction $s_{2}$ to the single-particle entropy $s_{1}$ yields values that are close to experiment but lie slightly below, as is evident in Figure 1 (blue triangles). However, we know that liquid metals have an electronic entropy (see Section 3.3), $S_{\mathrm{Elec}}$, and when we include that term (Figure 1, orange crosses) the values lie within 1 J/K/mol of the experimental values. Had we chosen to add $s_{2}-1/2+S_{\mathrm{Elec}}$ to $s_{\mathrm{Ideal}}$ instead of adding $s_{2}+S_{\mathrm{Elec}}$ to $s_{1}$ the values would have been greater by R/2=4.157 J/K/mol, resulting in poorer agreement (Figure 1 magenta + signs). In Section 3.4 we explain why $s_{1}+s_{2}+\ldots$ is a more suitable starting point for an expansion in multiparticle correlation functions than $s_{\mathrm{Ideal}}+\left(s_{2}-1/2\right)+\ldots$ is.

### 3.3. Electronic Entropy

The electronic density of states D(E), which comes as a byproduct of first principles calculations, determines the electronic entropy [22]. At low temperatures, all states below the Fermi energy $E_{F}$ are filled and all states above are empty. At finite temperature, single electron excitations vacate states below $E_{F}$ and occupy states above, resulting in the Fermi-Dirac occupation function

(26) $$f_{T}\left(E\right)=\frac{1}{\exp\left[\left(E-\mu \right)/k_{B}T\right]+1}.$$

Fractional occupation probability creates an electronic contribution to the entropy,

(27) $$S_{\mathrm{Elec}}=-k_{B}\int D\left(E\right)\left[f_{T}\left(E\right)\ln f_{T}\left(E\right)+\left(1-f_{T}\left(E\right)\right)\ln1-f_{T}\left(E\right)\right].$$

We apply this equation to representative configurations drawn from our liquid metal simulations, with increased *k*-point density in order to converge the density of states.

At low temperatures, the electronic entropy approaches $\left(\pi^{2}/3\right)D\left(E_{F}\right)k_{B}^{2}T$, which depends only on the density of states at the Fermi level. However, at the high temperatures of liquid metals the electronic entropy requires the full integral as given in Equation (27), rather than its low temperature approximation.

### 3.4. Three- and Higher-Body Terms

We saw in Figure 5 that the integral in Equation (21) converges slowly to the dimensionless compressibility γ which is a positive but very small value. Accordingly, the same must be true for the integral of the three-body fluctuation term, Equation (24), and all higher-body terms as well. Thus all fluctuation terms are essentially negligible contributions to the entropy at the temperatures considered here. This observation must break down at sufficiently high temperatures, because in the limit of very high temperature all correlation functions approach 1, so that all integrals vanish. As noted by Baranyai and Evans [11], $s_{2}\to 1/2$, $s_{3}\to 1/6$, $s_{4}\to 1/12$ and $s_{3}+s_{4}+\cdots \to 1$. This limit only holds at extreme high temperatures and low densities, however, the small shortfall in $s_{1}+s_{2}+S_{\mathrm{Elec}}$ at T = 2473 K could reflect a need to include a small fluctuation contribution due to the many-body terms $S_{\mathrm{Fluct}}^{\left(n\right)}$ at high temperatures.

**Figure 6:**
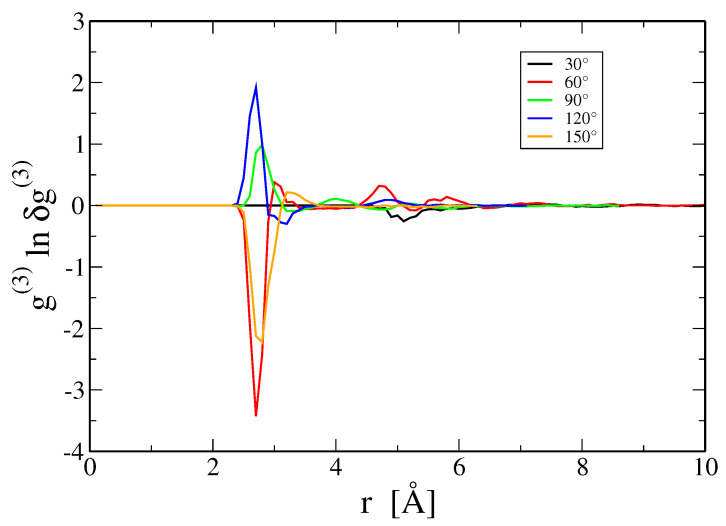
Three-body integrand $g^{\left(3\right)}\left(r,r,t\right)\ln\left(g^{\left(3\right)}\left(r,r,t\right)/g^{\left(2\right)}\left(r\right)g^{\left(2\right)}\left(r\right)g^{\left(2\right)}\left(t\right)\right)$ for isosceles triangles of angle θ and sides (r,r,t=2rcosθ) at T = 973 K.

We still need to discuss the three-body information term, $S_{\mathrm{Info}}^{\left(3\right)}$ (Equation (17)). Previous studies have discussed this term for model Lennard-Jones and hard-sphere fluids [23,24]. This term vanishes within the Kirkwood superposition approximation, $\delta g^{\left(3\right)}=1$, and as seen in Figure 4 this approximation is quite accurate even at T = 973 K. Presumably the nearly free electron character of aluminum, which causes its interactions to be well described by a nearly hard-sphere pair potential [25], leads to the weak form of $\delta g^{\left(3\right)}$. The deviations of $\delta g^{\left(3\right)}$ from 1 are oscillatory, both in radial dependence as seen in Figure 4, and in angle as shown in Figure 6. We lack sufficient resolution in $g^{\left(3\right)}$ to evaluate the complete integral, however, integrating over *r* at fixed angle the terms are of magnitude 0.1 or less, and they reverse sign as a function of angle, leading to further cancellation.

## 4. Discussion

We find that the entropy of liquid aluminum is described rather accurately using the first two terms in an expansion of the entropy in multiparticle correlations. We show in particular that it is advantageous to start the series with $s_{1}$ rather than $s_{\mathrm{Ideal}}$, and in compensation to include the terms 1/2, 1/6, … within $s_{\mathrm{Fluct}}^{\left(2\right)}$, $s_{\mathrm{Fluct}}^{\left(3\right)}$, …, respectively, because each of these terms then becomes of the order of the small dimensionless compressibility γ. The remaining terms, $S_{\mathrm{Info}}^{\left(n\right)}$, each have a simple information-theoretic interpretation, with $s_{1}$ being the information to specify individual particle positions with resolution $\Lambda^{3}$, $S_{\mathrm{Info}}^{\left(2\right)}=-I\left[g^{\left(2\right)}\right]$ being the mutual information content of the pair correlation function, and the corresponding higher order terms reflecting the additional information contained in $g^{\left(n\right)}$ that is not already present in the lower order terms.

In terms of accuracy, obtaining an accurate density is important. The difference between densities predicted at different system sizes *N* can shift the value of $s_{1}$ by about 0.5 J/K/mol, with greater density reducing $s_{1}$. More significant is the impact of density on $s_{2}$, with the same difference in density increasing the mutual information $I[g^{\left(2\right)}]$ by up to 6 J/K/mol. Both of these potential sources of error substantially exceed the truncation error due to neglect of multiparticle correlations, a finding that may hold generally for nearly-free-electron metals, while transition metals with angle-dependent forces may require additional terms.
